# Propensity score analysis in the Genetic Analysis Workshop 17 simulated data set on independent individuals

**DOI:** 10.1186/1753-6561-5-S9-S71

**Published:** 2011-11-29

**Authors:** Chen Min Lin, Fah J Sathirapongsasuti, Berit Kerner

**Affiliations:** 1 Department of Psychiatry, David Geffen School of Medicine, University of California, Los Angeles, 695 Charles E. Young Drive South, Los Angeles, CA 90095-1761, USA; 2 Department of Human Genetics, David Geffen School of Medicine, University of California, Los Angeles, 695 Charles E. Young Drive South, Box 708822, Los Angeles, CA 90095-7088, USA

## Abstract

Genetic Analysis Workshop 17 provided simulated phenotypes and exome sequence data for 697 independent individuals (209 case subjects and 488 control subjects). The disease liability in these data was influenced by multiple quantitative traits. We addressed the lack of statistical power in this small data set by limiting the genomic variants included in the study to those with potential disease-causing effect, thereby reducing the problem of multiple testing. After this adjustment, we could readily detect two common variants that were strongly associated with the quantitative trait Q1 (C13S523 and C13S522). However, we found no significant associations with the affected status or with any of the other quantitative traits, and the relationship between disease status and genomic variants remained obscure. To address the challenge of the multivariate phenotype, we used propensity scores to combine covariates with genetic risk factors into a single risk factor and created a new phenotype variable, the probability of being affected given the covariates. Using the propensity score as a quantitative trait in the case-control analysis, we again could identify the two common single-nucleotide polymorphisms (C13S523 and C13S522). In addition, this analysis captured the correlation between Q1 and the affected status and reduced the problem of multiple testing. Although the propensity score was useful for capturing and clarifying the genetic contributions of common variants to the disease phenotype and the mediating role of the quantitative trait Q1, the analysis did not increase power to detect rare variants.

## Background

Although genome-wide association studies in population samples may provide information on disease associations with relatively common genetic polymorphisms, they give only a tiny glimpse of the underlying functional variants and networks that might contribute to disease processes. Especially, rare variants in gene coding regions that are not in linkage disequilibrium with common variants are not well represented in these studies. Therefore exome resequencing projects have emerged to fill this knowledge gap; these studies focus on the detection of rare variants in coding regions of the genome. In addition, phenotypes are often influenced by many underlying quantitative traits and environmental exposures that might contribute to disease risk. These factors might be present in case subjects as well as in control subjects, therefore complicating the detection of risk factors. Covariates might introduce bias into the analysis if they are not evenly distributed between case subjects and control subjects. As confounding factors they might also obscure the relationship between risk factors and disease. New statistical approaches are clearly needed to address these analytical challenges.

Propensity score analysis is a relatively novel statistical approach to dimension reduction, bias detection, and risk estimation in studies where multiple covariates are present [1]. In clinical trials, the propensity score is often understood as the conditional probability of being assigned to a “treatment” group given the subject’s observed characteristics. It can be used to balance confounding covariates in the treatment and control groups, therefore reducing the selection bias in observational studies [2].

Three major applications of the propensity score have emerged. In the first approach the propensity score is used to reduce bias in observational case-control analyses if covariates are not evenly distributed between the case and control groups. In this context, case and control subjects could be matched one to one on the propensity score to create more homogeneous groups for comparison. This approach often leads to a significant reduction in sample size through elimination of unmatched cases [3]. In the second approach based on the propensity score, strata can be created to allow for multiple matches and retention of larger sample sizes if perfect matches cannot be found [4]. Finally, in the most common application, the propensity score is used to summarize information on confounding covariates into a single score, and then the propensity score itself is included as a covariate in a logistic regression model predicting the outcome [5].

In this study, we explore the use of the propensity score as a means to reduce the multiple dimensions of the phenotype in a case-control design using the exome sequencing data provided in the framework of the Genetics Analysis Workshop 17 (GAW17). Because the outcome is directly influenced by multiple quantitative traits, we use the propensity score to summarize the information on the covariates and to create a new quantitative trait or outcome variable, the probability of being affected given the covariates. Then, we compare this multivariate approach with the univariate approach in a case-control association analysis after reducing the multiple-testing problem even further by selecting only potential disease-causing genomic variants.

## Methods

### Sample

In the framework of GAW17, exome data for 697 unrelated individuals, a simulated phenotype, and environmental exposure were provided to explore and compare analytical methods for the detection of rare variant/disease associations [6]. For this analysis we use the replicate data set UNR_PH1. Information on the phenotype includes sex, age, ethnicity, three normally distributed quantitative traits related to the disease status (Q1, Q2, and Q4), and the affected status itself, coded as a binary variable. Information on smoking status is given as environmental exposure. The disease phenotype was created using a liability threshold model, and the disease prevalence increases with age. The top 30% with the highest liability (209 subjects) were assigned the affected phenotype, and the remaining 488 subjects were used as control subjects. The phenotype variables and environmental exposure were present in both case and control subjects and were unevenly distributed between the two groups (Table 1). Seven major ethnic subpopulations are present in this data set: Caucasians from the CEPH families (European-descended residents of Utah), Chinese from Denver, Han Chinese, Japanese, Luhya, Tuscans, and Yoruba. Each ethnic group contributed between 10% and 16% of the data. The affected status was fairly evenly distributed among the different ethnicities with an overall frequency between 30% and 50%.

**Table 1:** Distribution of covariates between case and control subjects

Covariate	Case group (*N* = 209)				Control group (*N* = 488)			
	
	%	*N*	Mean	SD	%	*N*	Mean	SD
Sex = male	45.0	94			47.8	233		
Smoking	35.9	75			21.7	106		
Q1			0.91	0.80			−0.39	0.81
Q2			0.59	0.99			−0.25	0.89
Q4			−0.80	0.99			0.34	0.78

### Propensity score analysis

**Figure 1:**
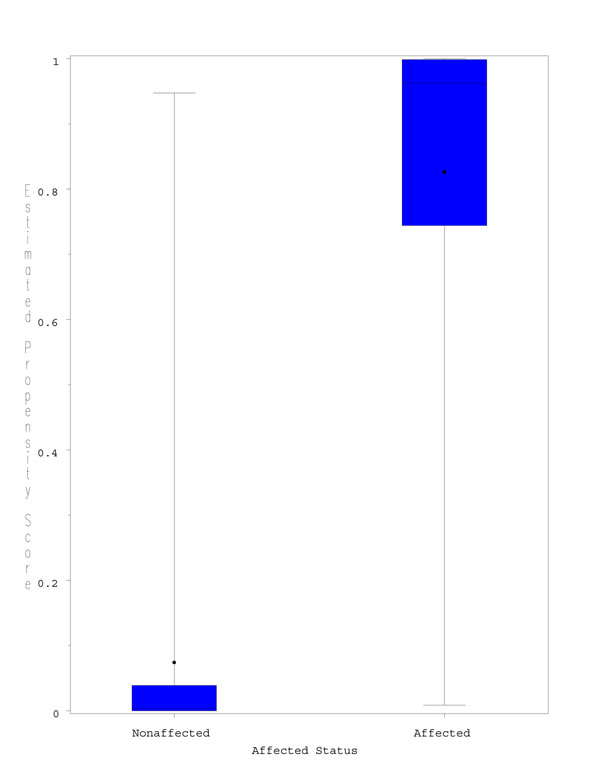
**Boxplots of the estimated propensity scores.** The boxplots show the distribution of the estimated propensity scores in the affected and nonaffected groups, as defined in the original data set. The whiskers indicate 1.5 times the interquartile range.

We use the propensity score to summarize the disease-related covariates as the predicted probability (*p*) of being affected given the covariates (Figure 1). The conditioning variable selection for the propensity score analysis was performed using stepwise backward regression. We used the covariates Q1, Q2, Age, Sex, Ethnicity (coded as integer), and Smoking as predictors and the binary affection status as the response variable in the stepwise backward regression. Q4 was found to be a protective factor. The four risk factors (Q1, Q2, Age, and Smoking) significantly influenced the affected status. Because Age did not have a genetic risk factor, we excluded it from the propensity score calculation. The selected variables were then included in the calculation of the propensity score using probit regression in Stata 11 (Table 2). In the propensity score estimation, the dependent variable was the log odds of being affected, and the conditioning variables (*X*) were considered the independent variables. The propensity score *e*(*x_i_*) for subject *i* (*i* = 1, …, 697) was defined as the conditional probability of being affected (*W_i_* = 1, case) versus nonaffected (*W_i_* = 0, control) given a vector of observed covariates *x_i_*:(1)

**Table 2:** Variable selection by stepwise regression

Response variable	Regressor	Coefficient	Standard error	Probability (chi-square test)	Odds ratio
Affected	Q1	2.32	0.25	1.22 × 10^−38^	10.22
	Q2	2.39	0.26	4.67 × 10^−39^	10.97
	Smoking	1.38	0.37	0.0001	3.99
	Age	0.14	0.01	2.50 × 10^−43^	1.15

### SNP selection

The genotype information was based on data from the pilot3 study of the 1000 Genomes Project [7] provided in the framework of GAW17. Based on autosomal sequence data, 24,487 single-nucleotide polymorphism (SNP) genotypes located in 3,205 known genes were provided. We removed synonymous SNPs and SNPs of unknown function based on the assumption that these SNPs were most likely not disease related. Variants with minor allele frequencies (MAFs) less than 0.001 were also removed, because those SNPs were present in only one individual and disease association would have been difficult to assign under these circumstances. The remaining 8,079 nonsynonymous SNPs were retained for further analysis.

### Genome-wide association analysis

Initially, we performed a univariate genome-wide case-control association analysis, first on the binary affected status and then on the quantitative traits Q1, Q2, and Q4 individually using the correlation trend test under the additive genetic model as implemented in the software program SVS, version 7, from Golden Helix. Population stratification present in this data set was corrected with 10 principal components, as indicated by the scree plot. Then, we used the propensity score, defined as the probability of being affected given the contributing covariates, as the outcome variable in the case-control association analysis. The Bonferroni approach was used to correct for multiple testing. In addition, we performed 10,000 single-value permutations and full-scan permutations in SVS, version 7, to confirm our results [8].

## Results

In the case-control association analysis, limitations and advantages of the different approaches became readily apparent. Selecting only nonsynonymous SNPs that were present in at least two individuals in the case or control group increased the power of our analysis by reducing the multiple testing issues. Following this approach, quantitative trait analysis for Q1 detected strong and significant associations with two SNPs: C13S523 (Bonferroni-corrected *p* = 7.1 × 10^−9^, permutation *p* = 0.0001) and C13S522 (Bonferroni-corrected *p* = 4.8 × 10^−7^, permutation *p* = 0.0001) (Table 3). Both SNPs had moderate MAFs (0.07 for C13S523 and 0.03 for C13S522) and a high *β* value (0.6). The correlation between Q1 and the propensity score was 0.67 (*p* < 0.0001). No significant associations were found with the affected status itself or with any of the other quantitative traits after correction for multiple testing.

**Table 3:** Genome-wide association analysis on univariate and multivariate phenotypes

Phenotype	SNP	Correlation trend test *p*-value	Bonferroni-corrected *p*-value	Permutation *p*-value	MAF
Affected	–	–	–		–
Q1	C13S523	8.8 × 10^−13^	7.1 × 10^−9^	0.0001	0.07
	C13S522	6.0 × 10^−11^	4.8 × 10^−7^	0.0001	0.03
Q2	–	–	–		–
Q4	–	–	–		–
Propensity score	C13S523	2.3 × 10^−9^	1.8 × 10^−5^	0.0001	0.07
	C13S522	2.3 × 10^−7^	0.001	0.0001	0.03

Case-control association analysis with the propensity score as the quantitative trait correctly identified the association with C13S523 (*p* = 2.3 × 10^−9^, Bonferroni-corrected *p* = 1.8 × 10^−5^) and C13S522 (*p* = 2.3 × 10^−7^, Bonferroni-corrected *p* = 0.0018) mediated through Q1 (Table 3). Permutation analysis with 1,000 permutations revealed a permutation *p* = 0.001 for both associations. Both SNPs were located in the gene *FLT1*. Even though we were able to capture the correlation between Q1 and the affected status and to detect true associations with common variants (no false-positive signals were found at the genome-wide level of significance), our approach was unable to detect additional rare variants. The analysis missed 69 true disease-related SNPs with MAFs between 0.17 and 0.00071, and *β* values between 1 and 0.03, including nine additional SNPs in the gene *FLT1*.

A common approach to rare genomic variants is the selection of variants that are present only in case subjects. Taking this approach, we found 421 SNPs that fulfilled this criterion; 16 of those were present in only one case subject. Out of 405 SNPs present in at least two case subjects, only 5 SNPs in five different genes were associated with disease according to the model and 400 SNPs were false positives. The most obvious contributing factor to the inflation of rare variants is ethnic admixture in this data set. Even though we attempted to correct for this problem using principal components analysis, it could not be completely eliminated.

## Discussion

Composite phenotypes that are strongly influenced by multiple quantitative traits with specific genetic risk factors are a frequently encountered phenomenon in genetic studies of common complex disorders. Often these traits are present in case subjects as well as in control subjects, and they might introduce bias or even be confounding factors in the estimates of disease associations. A joint estimate of these covariates is rarely included in genome-wide association studies.

Propensity score analysis has emerged as an approach to dimensionality reduction. Because the contributing quantitative traits are present in both case and control subjects, a probabilistic approach that summarizes the multiple risk factors is appropriate, particularly because the genetic risk factors predominantly influence the affected status through the quantitative traits. Using propensity score analysis, we were able to detect two SNPs associated with the affected phenotype that were obscured when only the affected phenotype was used as the outcome, and we were able to clarify the relationship between the quantitative traits and the phenotype.

The application of the propensity score in the more traditional sense as a means to reduce bias was limited in this data set. After all, potentially confounding covariates were not the focus of this simulation. In fact, most covariates were directly and causally related to the affected status. Attempts to match case and control subjects on the propensity score either by one-to-one matching or by stratification resulted in a significant reduction in sample size as a result of a large number of unmatched observations. The resulting loss of power made the detection of significant associations impossible. Using the propensity score as a covariate after inclusion of all known contributing variables eliminated all the genetic contributions to the affected status mediated by the quantitative traits.

Still, using the propensity score as a dimensionality reduction tool has several advantages over multivariate regression. Multivariate regression models are often concerned with finding parsimonious models using only a limited number of covariates to avoid overparameterization. In the propensity score estimation, the number of covariates that can be included is not limited by the model. Interactions and nonlinear terms can easily be incorporated.

A commonly encountered problem in case-control association studies is false-positive association resulting from nonrandom differences between case and control subjects that are not related to the presence of the disease itself [9,10]. Population stratification resulting from admixture of different ethnic groups with differences in allele frequencies or uneven distribution of sex and other confounding covariates can introduce biases that are frequently not addressed in the study design. In the GAW17 data set, ethnicity was such a confounding factor. Ethnicity itself was not related to the disease status; however, the presence of seven different ethnicities introduced a large number of rare and private mutations in the data set. The overwhelming number of rare and private variants that were not related to disease in only the case group cautions against the assumption in studies involving independent individuals and complex disorders that presence in case subjects and absence in control subjects is evidence of pathogenicity. This data set demonstrates that ignoring design issues, particularly population admixture and unbalanced covariates between case and control subjects, can introduce noise into the data and can complicate the discovery of true disease associations. Post hoc statistical analysis cannot always correct for these design issues.

Sequencing data sets still have a relatively small sample size that limits the power of a study to detect associations with rare variants in a traditional case-control design. Therefore, it might be useful to identify genomic variants that are more likely to cause disease, such as nonsynonymous variants and truncating and non-sense mutations in coding regions, through resequencing approaches. Focus on those variants would decrease the multiple testing problem and increase the power to detect disease-associated variants with large effect. However, realistic expectations should be in place when dealing with these data. Case-control association designs might not be the appropriate approach to rare variants, particularly under genetic heterogeneity. Family-based resequencing approaches might be more appropriate under these conditions.

## Conclusions

Propensity score analysis could be a useful tool in genetic case-control association analyses. Even though we admit that this simulated data set had limitations for the meaningful use of this method, our study demonstrates an application to the dimensionality reduction of phenotypes that are influenced by multiple correlated traits with strong genetic risk factors. This approach might give some advantage in settings in which issues related to multiple testing arise. Potential problems include the selection of covariates. Our approach did not increase the power to detect rare variants, which remains a problem that is difficult to address in case-control studies.

## Competing interests

The authors declare that there are no competing interests.

## Authors’ contributions

CML performed the propensity score analysis and created the figure. JFS performed the regression analysis for the variable selection and the regression analysis using propensity scores as co-variates. BK performed the genetic case-control association analysis, supervised the work of CML and JFS, and wrote the paper. All authors participated in the study design, the discussion and interpretation of the results. All authors have read and approved the final manuscript.

## References

[B1] Rosenbaum PR Rubin DB The central role of the propensity score in observational studies for causal effects Biometrika 1983 70 41 55 10.1093/biomet/70.1.41

[B2] Weitzen S Lapane KL Toledano AY Hume AL Mor V Principles for modeling propensity scores in medical research: a systematic literature review Pharmacoepidemiol Drug Saf 2004 13 841 853 10.1002/pds.969 15386709

[B3] Stuart EA Rubin DB J Osborne Best practices in quasi-experimental designs: matching methods for causal inference Best Practices in Quantitative Social Science 2007 Thousand Oaks, CA, Sage 155 176

[B4] Stuart EA Green KM Using full matching to estimate causal effects in nonexperimental studies: examining the relationship between adolescent marijuana use and adult outcomes Dev Psychol 2008 44 395 406 18331131 10.1037/0012-1649.44.2.395 PMC5784842

[B5] Guo S Fraser MW Propensity Score Analysis: Statistical Methods and Applications 2010 Los Angeles, CA, Sage 127 210

[B6] Almasy LA Dyer TD Peralta JM Kent JW Jr Charlesworth JC Curran JE Blangero J Genetic Analysis Workshop 17 mini-exome simulation BMC Proc 2011 5 suppl 9 S2 22373155 10.1186/1753-6561-5-S9-S2 PMC3287854

[B7] 1000 Genomes Project Consortium Durbin RM Abecasis GR Altshuler DL Auton A Brooks LD Durbin RM Gibbs RA Hurles ME McVean GA A map of human genome variation from population-scale sequencing Nature 2010 467 1061 1073 10.1038/nature09534 20981092 PMC3042601

[B8] SNP and Variation Suite™ Manual, Version 7.4.0 Permutation Testing Methodology, Goldenhelix, Inc. 6/23/2011 http://www.goldenhelix.com/SNP_Variation/Manual/svs7/manual.html

[B9] Zhao H Rebbeck TR Mitra N A propensity score approach to correction for bias due to population stratification using genetic and non-genetic factors Genet Epidemiol 2009 33 679 690 10.1002/gepi.20419 19353632 PMC4537699

[B10] Wu CK Luo JL Wu XM Tsai CT Lin JW Hwang JJ Lin JL Tseng CD Chiang FT A propensity score-based case-control study of renin-angiotensin system gene polymorphisms and diastolic heart failure Atherosclerosis 2009 205 497 502 10.1016/j.atherosclerosis.2008.12.033 19185300

